# Case Report: *De novo* Vertebral Artery Dissection After Intravascular Stenting of the Contralateral Unruptured Vertebral Artery Aneurysm

**DOI:** 10.3389/fneur.2021.599197

**Published:** 2021-04-23

**Authors:** Wei You, Junqiang Feng, Qinglin Liu, Xinke Liu, Jian Lv, Yuhua Jiang, Peng Liu, Youxiang Li

**Affiliations:** Department of Interventional Neuroradiology, Beijing Neurosurgical Institute, Beijing Tian Tan Hospital, Capital Medical University, Beijing, China

**Keywords:** vertebral artery dissecting aneurysm, *de novo* aneurysm, bilateral vertebral artery dissection, endovascular embolization, pipeline embolization device

## Abstract

Spontaneous vertebral artery dissecting aneurysm has been increasingly attributed as a major cause of focal neurological deficits due to vertebrobasilar artery ischemia or subarachnoid hemorrhage (SAH). Although the development of spontaneous vertebral artery dissecting aneurysm (VADA) is rare, *de novo* VADA after treatment of contralateral vertebral artery (VA) is more less frequently observed. There are only a few reports related to *de novo* VADA after treatment of the contralateral VA in the medical literature. The mechanisms responsible for *de novo* dissection after treatment of unilateral VADA are still not clearly understood. In this manuscript, we report an unusual case of a patient with a *de novo* VADA after placement of a pipeline embolization device (PED) stent on the contralateral VA along with a thorough review of the literature. A 42-years old male patient was referred to the hospital with sudden onset of dizziness, nausea, and vomiting. Initial digital subtraction angiography (DSA) images demonstrated a VADA in the fourth segment of the left VA without the involvement of the posterior inferior cerebellar artery (PICA). There were no significant abnormalities found in the right vertebral artery. He underwent an endovascular pipeline embolization to treat the dissecting aneurysm (DA). Surprisingly, follow-up DSA imaging 14 months after the initial treatment showed a segmental dilatation and narrowing of the right VA, which suggested a *de novo* VADA on the right side that had occurred postoperatively. This was followed by a tent-assisted coil embolization therapy for occluding this *de novo* VADA. This patient showed an uneventful postoperative course with no neurological abnormalities. In addition to hemodynamic stress changes, the unique clinicopathological features of dissecting aneurysms may contribute significantly to the pathogenesis of *de novo* VA dissection. Given that VA in VADA patients may be vulnerable on both sides, it is important to consider the risk of *de novo* dissection after initial aneurysm treatment. The bilateral vertebral artery has to be carefully observed when treating any VADA patient to prevent any complications.

## Introduction

With a significant improvement in the understanding of the disease entity and angiographic appearance, the vertebral artery dissecting aneurysm (VADA) is considered rare, but has been increasingly reported as a fairly common cause of subarachnoid hemorrhage (SAH) or brain stem ischemia (1). In cases with SAH, previous studies have reported a high incidence of rebleeding with a high mortality rate during the time of recurrent bleeding (2, 3) thereby underscoring the necessity of early interventions. The development of spontaneous vertebral artery (VA) dissecting aneurysm is of rare occurrence, and *de novo* VADA after treatment of contralateral VA has been even less commonly observed. Mechanisms underlying *de novo* dissection after treatment of unilateral VADA have not been completely deciphered. In this manuscript, we report an unusual case of a patient with a *de novo* VADA after placement of a PED stent on the contralateral VA followed by an exhaustive literature review.

## Case Description

**Figure 1:**
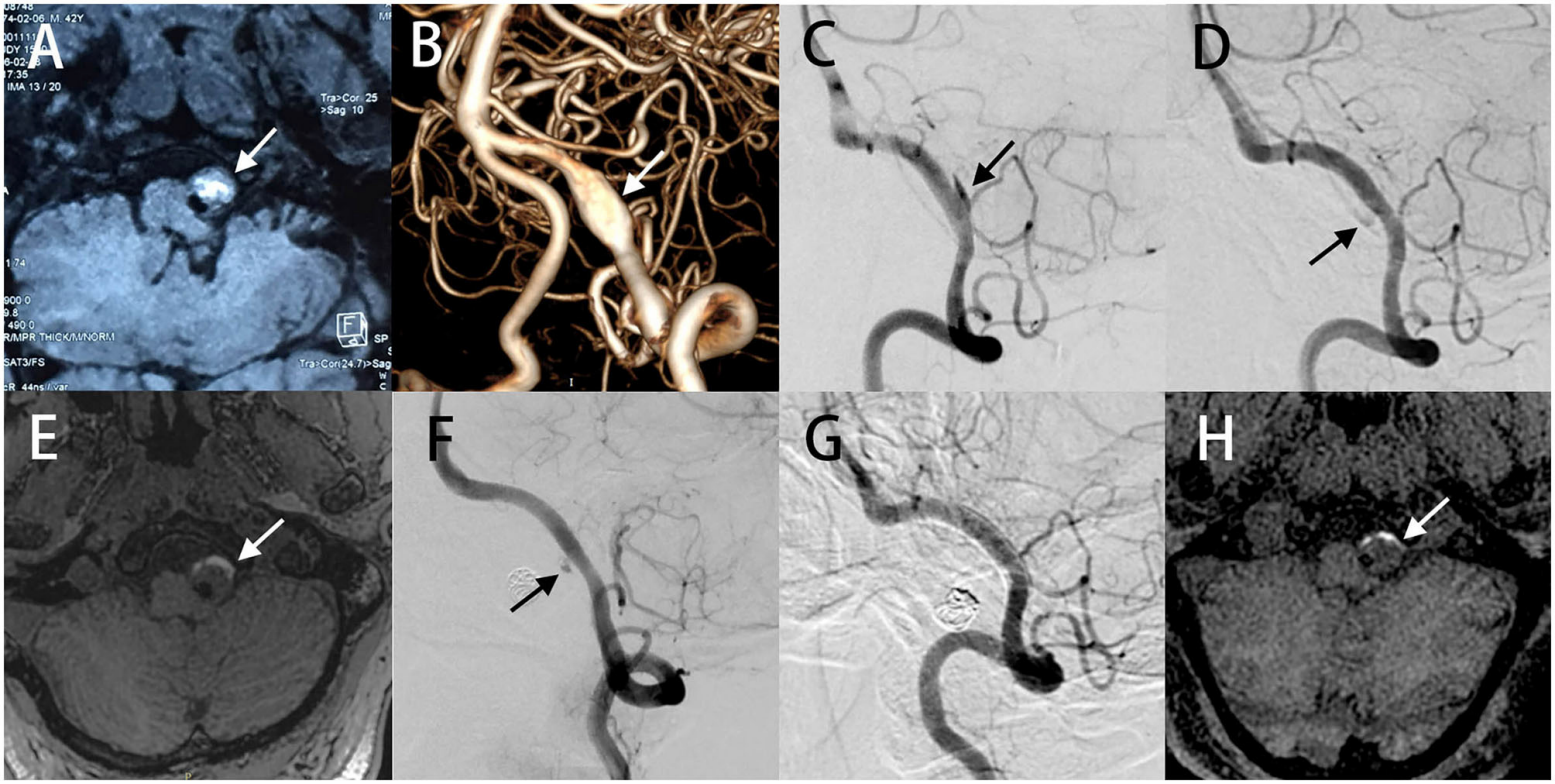
MR image detected a partially thrombosed aneurysm adjacent to the left part of medulla **(A)**. 3D DSA image demonstrated a VADA in the fourth segment of the left VA **(B)**. Angiography at 5 months follow-up revealed that the stent were patent with partial aneurysm residues **(C)**. Partial residual aneurysm of the left VADA was observed on 14 months **(D)** and 2 years **(F)** follow-up angiography, and complete occlusion on 3 years (G). The volume of the aneurysm was not significantly changed from 14 months **(E)** and 2 years follow-up MR images **(H)**.

A 42-years old male patient was referred to a local hospital with a sudden onset of dizziness, nausea, as well as vomiting, and MRI revealed a partially thrombosed aneurysm adjacent to the left portion of the medulla (Figure 1A). The patient was admitted to our hospital without any major symptoms. An initial DSA image demonstrated dilatation at the fourth segment of the left VA, thus indicating a VADA without the involvement of the posterior inferior cerebellar artery (PICA) (Figure 1B). The right vertebral and basilar artery showed no major abnormalities. He had a medical history of hypertension and hyperlipidemia, but no previously reported head trauma and family history of aneurysm. The patient had a history of smoking 20 cigarettes a day for 20 years, which was ceased just at the time of this admission.

We treated the left VADA using endovascular pipeline embolization for preserving the normal blood flow. In addition, a dual antiplatelet therapy, comprising 300 mg aspirin and 300 mg clopidogrel were administered 5 days before the surgery. Under general anesthesia, a pipeline embolization device (PED) was successfully implanted with satisfactory adherence between the PED and vessel wall. No intraoperative complications were encountered, and the right VA was preserved. He was discharged home 1 week after the operation and prescribed dual antiplatelet therapy (aspirin 100 mg/day and clopidogrel l00 mg/ day) for 6 months. Angiography conducted at 5 months after initial treatment revealed the patency of the VA and partial aneurysm residues (Figure 1C). This residual of the left VADA persisted on 14 months (Figure 1D) and 2 years (Figure 1F), and completely occluded at 3 years angiography follow-up (Figure 1G). The volume of the aneurysm did not significantly alter from 14 months (Figure 1E) and as noted in 2 years of follow-up MR images (Figure 1H), and the patient did not display any adverse symptoms after the surgery.

**Figure 2:**
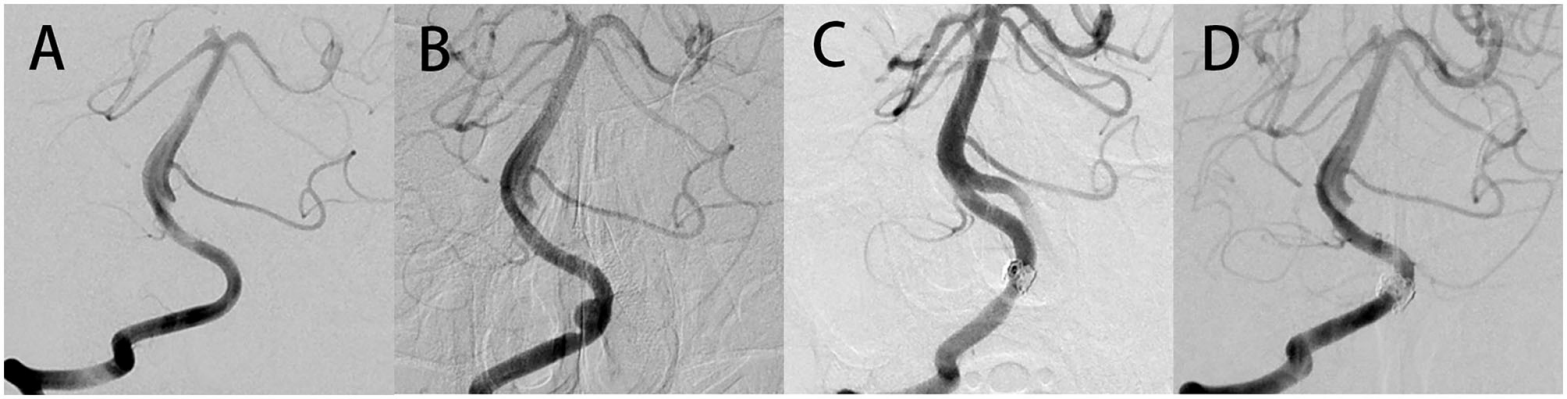
No abnormalities were found in the right VA at the time of 5 months after initial treatment **(A)**. Follow-up imaging 14 months postoperatively showed a segmental dilatation and narrowing of the right VA, which suggested that a *de novo* VADA occurred **(B)**. Stent-assisted coil embolization therapy was performed to occlude this *de novo* VADA **(C)**. Angiography at 20 months follow-up after surgery revealed a complete occlusion of the aneurysm **(D)**.

In addition, no major abnormalities were found in the right VA at 5 months after initial treatment (Figure 2A). Surprisingly, follow-up imaging 14 months postoperatively showed a segmental dilatation and narrowing of the right VA (Figure 2B), which suggested the formation of a *de novo* VADA. Stent-assisted coiling was performed for this *de novo* VADA. Under general anesthesia, Guglielmi detachable coils were positioned in the dissecting aneurysms after placing an LVIS stent in the true lumen of the VA (Figure 2C). Dual antiplatelet therapy was prescribed to him as done before. The patient had an uneventful postoperative progression with no observation of any occurrence of neurological deficits. Moreover, a 20 months angiography follow-up revealed complete occlusion of the aneurysm (Figure 2D).

## Discussion

Dissecting aneurysms of the intracranial vertebral arteries are observed rarely, and either present themselves as ischemic symptoms of the brain stem or subarachnoid hemorrhage (SAH) (4–6). Unruptured dissecting aneurysms typically may have a benign course, and conservative measures such as antiplatelet or anticoagulation therapy are often recommended (6). However, once ruptured, vertebrobasilar aneurysms may have a poor prognosis with a mortality rate of approximately 50%, and recurrent hemorrhage can account for between 24 and 70% (4–6). Hence, appropriate treatment modalities are needed to avoid serious complications. When treating a VADA, a suitable consideration should be given to angioarchitecture including VA dominancy, location of the PICA origin, and anterior spinal artery involvement. Given its minimally invasive characteristics, endovascular treatment of VADA has become one of the most commonly used method, including internal trapping and stenting. Although internal trapping was the previously preferred treatment, with the advent of the appropriate use of antiplatelet agents and newly developed flow diverters, stenting has also shown favorable safety and efficacy in the management of VADA. Therefore, stent implantation was performed on the left VADA to maintain normal blood flow. The aneurysm reached complete occlusion at 3 years follow-up angiography. Numerous studies (7, 8) have revealed shrinkage of aneurysms following PED placement in cerebral aneurysms, however, the volume of the aneurysm in the present case was not significantly altered from follow-up MR images. The presence of substantial prior thrombosis appears to compromise the reduction of aneurysm volume after FD treatment.

Surprisingly, a follow-up angiography at 14 months after the initial treatment revealed a *de novo* VADA in the right vertebral artery, which is a very interesting observation. However, there is a paucity of data related to the *de novo* aneurysm formation rates in different patients with unruptured aneurysms. Moreover, in a systematic review and meta-analysis involving nearly 15,000 patients, the incidence of *de novo* aneurysms in patients with unruptured aneurysms was observed to be around 3% (9). In addition, history of smoking, hypertension, family history, and female gender are considered as high-risk factors for the development of *de novo* aneurysms (9, 10).

There are only a few reports about the *de novo* VADA after treatment of the contralateral VA in the existing literature. Previously reported cases are summarized in Table 1 (11–17). Most initial aneurysms appear on the left side and manifest as SAH or infarction, and can be treated by trapping or occlusion of VA. The interval between the initial dissection and the discovery of *de novo* contralateral dissection varies from patient to patient. The mechanism responsible for *de novo* dissection after treatment of unilateral VADA has not been well-defined. It is however possible, that the unique clinicopathological features of dissecting aneurysms and changes in hemodynamic stress may significantly contribute to the pathogenesis of *de novo* VA dissection.

**Table 1:** A summary of case reports of *de novo* VADA after the treatment of the contralateral VA.

**References**	**Age (years)/Sex**	**Initial VADA**			**Interval**	**Second VADA**			**Outcome**
		**Location**	**Presentation**	**Treatment**		**Location**	**Presentation**	**Treatment**	
Kubo et al. (11)	49/F	L	SAH	Proximal occlusion	3 W	R	Asymptomatic	Proximal occlusion	GR
Otawara et al. (13)	51/F	R	SAH	Surgical trapping	1 Mon	L	Asymptomatic	Conservation	GR
Inui et al. (14)	36/M	L	Infraction	Conservation	12 Mon	R	Infraction	Conservation	Dead
	45/M	L	SAH	Endovascular trapping	2 W	R	Infraction	Conservation	SD
Katsuno et al. (15)	39/M	L	SAH	Surgical trapping	8 H	R	SAH	Conservation	Dead
Kidani et al. (17)	55/F	L	SAH	Endovascular trapping	3 Mon	R	Asymptomatic	Conservation	GR
Tsuji et al. (16)	52/M	L	Infraction	Conservation	9 D	R	SAH	Endovascular trapping	GR
Present 2020	42/M	L	Asymptomatic	Endovascular stenting	14 Mon	R	Asymptomatic	SAC	GR

*F, female; M, male; L, left; R, rightl; SAH, subarachnoid hemorrhage; W, week; Mon, month; H, hour; D, day; SAC, Stent-assisted coil; GR, good recovery*.

A few other studies suggest that sudden changes in hemodynamic stress may be the major causal factor behind the development of VA dissecting aneurysms. Two different cases have reported that the diameter of the VA increased after trapping of the contralateral VA (14, 18). Kono et al. performed the computational fluid dynamics (CFD) simulations of bilateral VADA and found that trapping of unilateral VA increased the wall shear stress in the dome surface of the contralateral aneurysm (19). Abrupt changes in hemodynamic stress after occlusion of unilateral VA may play an important role in the occurrence of contralateral VADA. However, as compared to the previously reported cases, our case retains the normal blood flow of unilateral VA, which may greatly alleviate the impact on hemodynamic changes. Furthermore, we noted that our patient had an uneventful postoperative with no neurological deficits and displayed good blood pressure control. Hemodynamic analysis by CFD can also aid in evaluating the formation and growth of aneurysms (20, 21), but there are few data available related to the correlation between hemodynamic changes after stenting and the occurrence of contralateral VADA (19). To the best of our knowledge, this is the first case of development of a *de novo* VADA after stent placing of the contralateral VA while the contralateral VAs blood flow was maintained in a normal manner.

The clinicopathological features associated with the intracranial dissecting aneurysms have been discussed in detail previously (22–24). The characteristic pathological features include defect or fragmentation of the internal elastic lamina, intimal thickening, and medial degeneration, which can lead to the formation of an aneurysm with or without relevant narrowing of the arterial lumen (24, 25). Generally, the main mechanism associated with intracranial arterial dissection is the diversion of the arterial stream into a weakened arterial wall. An important factor in this process is the development of multiple intramural hemorrhages, which are usually isolated and non-contiguous in the walls of the VA (22, 23, 26). However, these small intramural hemorrhages may be closely related to the disruption of vasa vasorum or new vessels. Although the pathogenesis and clinical manifestations of vertebral artery dissection and carotid artery dissection have not been fully explained, it is reported that patients with spontaneous intracranial artery dissection involve multiple arteries, and the incidence of spontaneous multivessel dissection has been found to be between 10 and 15% (27, 28). For example, Aronov et al. (29) reported a case of acute three-vessel carotid artery occlusion due to spontaneous quadruple carotid dissection occurring 1 week after cesarean section. Ro et al. (30) conducted a detailed pathological investigation of bilateral vertebral arteries in patients who died of SAH due to VADA. They found that 25 of the 58 patients had a latent previous dissection at a different location from the rupture point, with small disruption in the internal elastic lamina covered by an intimal thickening. Besides, they observed that the latent previous dissection had a tendency to occur as bilateral multiple lesions, thereby suggesting that the VA of patients with VADA may be vulnerable on both sides. It is unclear whether the *de novo* VADA, in this case, developed because of an extension of a latent previous dissection or by the occurrence of a possible new dissection. Therefore, the bilateral vertebral artery needs to be carefully observed when treating any VADA patient.

Many studies have suggested that smoking is a major risk factor for the formation of *de novo* aneurysms due to its propensity to result in an elastase/alpha antitrypsin imbalance, which may exacerbate the effect of hemodynamic stress on the aneurysm wall (31). Moreover, the other authors have speculated that hypertension may be a risk factor because the interval between identifying newly formed aneurysms has been noted to be significantly shorter in patients with hypertension (32). Furthermore, both smoking and hypertension may contribute to the degradation of the vessel wall and can lead to the development of *de novo* aneurysms as found in the present case.

It is worth mentioning that a recent Japanese survey of spontaneous cerebral arterial dissection showed that intracranial VA dissection can occur more frequently in Japan (33). This is completely different from the findings among the American population, which displayed a higher incidence of cervical internal carotid artery dissection (34). Actually, all previous case reports about *de novo* VA dissection were collected from Japan. The reason for this difference has not been clearly elucidated so far and may be possibly related to the variation in genes and the environment.

## Conclusions

Endovascular treatment with stent placement can often preserve the normal blood flow of the VA and thereby reduce the changes observed in postoperative hemodynamic stress, but there is still a substantial risk of *de novo* dissection. In addition to hemodynamic stress changes, the unique clinicopathological features of dissecting aneurysms may significantly contribute to the pathogenesis of *de novo* VA dissection. As VA in VADA patients may be at risk on both sides, the bilateral vertebral artery needs to be carefully monitored while treating VADA patients.

## Data Availability Statement

The original contributions presented in the study are included in the article/supplementary material, further inquiries can be directed to the corresponding author/s.

## Author Contributions

WY wrote the manuscript and edited the figure and the table of the article. Together with QL, XL, and JF performed the revision of the current literature. WY and JL collection and interpretation of patient data. YL, YJ, and PL conceived and designed the research. All authors contributed to the article and approved the submitted version.

## Conflict of Interest

The authors declare that the research was conducted in the absence of any commercial or financial relationships that could be construed as a potential conflict of interest.

## Abbreviations

SAH: subarachnoid hemorrhage
VA: Vetebral artery
DA: dissecting aneurysm
VADA: vertebral artery dissecting aneurysm
PED: pipeline embolization device
PICA: posterior inferior cerebellar artery
DSA: digital subtraction angiography
CFD: computational fluid dynamics.
